# Structural and functional connectional fingerprints in mild cognitive impairment and Alzheimer’s disease patients

**DOI:** 10.1371/journal.pone.0173426

**Published:** 2017-03-23

**Authors:** Seong-Jin Son, Jonghoon Kim, Hyunjin Park

**Affiliations:** 1 Department of Electronic, Electrical and Computer Engineering, Sungkyunkwan University, Suwon, Korea; 2 Center for Neuroscience Imaging Research, Institute for Basic Science (IBS), Suwon, Korea; 3 School of Electronic and Electrical Engineering, Sungkyunkwan University, Suwon, Korea; University of Pennsylvania Perelman School of Medicine, UNITED STATES

## Abstract

Regional volume atrophy and functional degeneration are key imaging hallmarks of Alzheimer’s disease (AD) in structural and functional magnetic resonance imaging (MRI), respectively. We jointly explored regional volume atrophy and functional connectivity to better characterize neuroimaging data of AD and mild cognitive impairment (MCI). All data were obtained from the Alzheimer's Disease Neuroimaging Initiative (ADNI) database. We compared regional volume atrophy and functional connectivity in 10 subcortical regions using structural MRI and resting-state functional MRI (rs-fMRI). Neuroimaging data of normal controls (NC) (n = 35), MCI (n = 40), and AD (n = 30) were compared. Significant differences of regional volumes and functional connectivity measures between groups were assessed using permutation tests in 10 regions. The regional volume atrophy and functional connectivity of identified regions were used as features for the random forest classifier to distinguish among three groups. The features of the identified regions were also regarded as connectional fingerprints that could distinctively separate a given group from the others. We identified a few regions with distinctive regional atrophy and functional connectivity patterns for NC, MCI, and AD groups. A three label classifier using the information of regional volume atrophy and functional connectivity of identified regions achieved classification accuracy of 53.33% to distinguish among NC, MCI, and AD. We identified distinctive regional atrophy and functional connectivity patterns that could be regarded as a connectional fingerprint.

## Introduction

Alzheimer’s disease (AD) is a chronic neurodegenerative disease characterized by a decline in cognitive function that results in problems with daily activities [1–3]. Mild cognitive impairment (MCI) is an intermediate stage between cognitively normal status and AD [4]. MCI involves problems with memory, language, thinking, and judgement that are greater than typical age-related changes, but the changes are not severe enough to interfere with daily life or independent function [4]. There is no generally agreed cure for far-progressed AD; thus, detecting MCI and AD early is important [3].

Subcortical regions, such as the thalamus, putamen, hippocampus, caudate, and amygdala, play a key role in MCI and AD [5]. These regions affect sensory, learning, motor, emotion, language, and memory. Regional volume atrophy is known to precede functional degeneration of those brain regions [6,7]. Our study also focused on these subcortical regions as well. Regional volume atrophy of cortical and subcortical structures was reported as an important imaging hallmark in AD [7–11].

Many imaging modalities, including magnetic resonance imaging (MRI), single photon emission tomography (SPECT), and positron emission tomography (PET), have been successfully adopted to evaluate the progression of MCI and AD [12–16]. MRI is especially advantageous as it can obtain both structural and functional information [17]. Resting-state functional MRI (rs-fMRI) reflects local brain activity using the blood oxygen level-dependent (BOLD) signal, which has been shown to be useful at distinguishing normal subjects, MCI, and AD patients [18,19]. Connectivity analysis treats the whole brain as a complex interconnected network, focusing on how activities in one region correlate with activities in another region [20–22]. Researcher can apply well-established theories and tools of network graphs with nodes and edges to quantify the connectivity of any given brain [21–24]. Centrality measure is used to assess the importance of a region (i.e., node) within a network [25]. Eigenvector centrality is one of several centrality measures that characterizes the prominence of a node in the network [26]. Eigenvector centrality can reflect both local and global characteristics of a given network [27].

Many studies have either employed a single modality (structural MRI or rs-fMRI) or measured global (i.e., not regional) connectivity properties in brains of AD or MCI patients [28–30]. A multi-modal neuroimaging study could take advantage of complementary information from different modalities and thus is better suited for analyzing AD and MCI brains. AD and MCI alter brain structure and function in a localized manner [31,32]. Thus, a study focusing on local properties of the brain is beneficial. Here, we employed multi-modal imaging to explore local properties of AD and MCI brains. A region might have a unique connectivity pattern that could be used to distinguish it from other regions [33]. A *connectional fingerprint* refers to the unique connectivity information of brain regions. Unique connectivity information can be derived from both functional MRI (via functional correlation) and structural MRI (via morphology) [33–35]. Recently, a connectional fingerprint has been used to distinguish between disease stages [34–36]. The changes in disease stage were reflected in an altered pattern of connectional fingerprint information.

In this study, we sought connectional fingerprint information using eigenvector centrality to identify a unique functional connectivity profile, and we used regional volume atrophy to identify unique morphological profile in AD and MCI patients. The rationale behind our study was to jointly explore regional atrophy and functional connectivity in subcortical regions for MCI and AD. Many existing studies only considered rs-fMRI to report functional connectivity change or structural MRI to report regional volume atrophy in subcortical regions to distinguish among NC, MCI, and AD [28–30]. We aimed to (1) identify regions with significant regional volume atrophy and eigenvector centrality that differ among NC, MCI, and AD groups; (2) apply a machine learning framework, random forest (RF) classifier, to distinguish between three patient groups using the identified regional information; and (3) present unique connectional fingerprints of NC, MCI, and AD based on volume atrophy and eigenvector centrality.

## Materials and methods

### Subjects and imaging data

This study was a retrospective analysis of anonymized data, and institutional review board (IRB) approval was obtained at Sungkyunkwan University. All data were obtained with informed written consent in accordance with established human subject research procedures expressed in Declaration of Helsinki. Our study was performed in full accordance with the local IRB guidelines. In this study, we obtained structural MRI and rs-fMRI images from the Alzheimer’s Disease Neuroimaging Initiative (ADNI) research database [37]. The sample consisted of 105 participants who were classified as normal control (NC) (n = 35), MCI (n = 40), and AD (n = 30) with matched age and sex ratios. The groups were classified according to the criteria set by the ADNI consortium [38]. In the NC group, patients had global clinical dementia rating (CDR) scores of 0 and mini-mental state examination (MMSE) scores between 24 and 30. In the MCI group, patients had global CDR scores of 0.5 and MMSE scores between 24 and 30. In the AD group, patients in the AD group had global CDR scores of 0.5 or 1.0 and MMSE scores between 20 and 26 [39,40]. Details regarding the patient groups, including age and sex ratios, are reported in Table 1. There were no significant differences (p-value > 0.05) between the comparison groups in age or sex ratio. All MRI images were obtained using a Philips medical system 3.0 T scanner. Structural MRI was performed with a T1-weighted magnetization-prepared rapid gradient-echo (MPRAGE) sequence with the following parameters: TR = 6.77 ms, TE = 3.13 ms, slices = 32, voxel size = 1 mm isotropic, and image size = 256×256×170 mm^3^. Rs-fMRI data were acquired with an EPI sequence with the following parameters: TR = 3,000 ms, TE = 30 ms, slices = 32, voxel size = 3.3125 mm isotropic, image size = 256×256×170 mm^3^, and number of time series = 140.

**Table 1:** Demographic data of the NC, MCI, and AD groups. Values are reported as mean (standard deviation).

	NC (n = 35)	MCI (n = 40)	AD (n = 30)
**Sex (M:F)**	12:23	19:21	12:18
**Age**	76.06 (7.38)	74.30 (7.67)	74.00 (7.46)
**CDR score**	0.04 (0.14)	0.54 (0.21)	1.02 (0.38)
**MMSE score**	29.43 (1.14)	27.55 (2.15)	19.40 (3.62)

NC, Normal controls; MCI, Mild cognitive impairment patients; AD, Alzheimer’s disease patients; M, Male; F, Female; CDR, Clinical dementia rating; MMSE, Mini-mental state examination

### Image pre-processing: Structural MRI

The pre-processing steps of structural MRI data were performed using the Athena pipeline that combines AFNI and FSL neuroimaging pipelines [41,42]. The raw T1 images underwent skull-stripping to remove non-brain tissue and background. The images were segmented into white matter (WM), cerebrospinal fluid (CSF), and grey matter (GM). An initial linear registration was performed between the skull-stripped image and standard Montreal Neurological Institute (MNI) structural template. The registration was subsequently refined by a non-linear registration procedure. The skull-stripped registered images were smoothed by a 6-mm full width at half maximum (FWHM) Gaussian filter.

### Image pre-processing: rs-fMRI

The rs-fMRI data were pre-processed using the Athena pipeline. The first four volumes were discarded to allow for magnetization to reach equilibrium. Slice timing was corrected to the middle slice, and each volume was re-aligned to the first volume to correct for motion. All volumes were linearly registered onto the corresponding T1 image [41,42]. The rs-fMRI onto T1 transform was then combined with the T1 to MNI non-linear registration to map the rs-fMRI images onto the MNI space of 4×4×4 mm^3^. Mean WM and CSF time courses were extracted using WM and CSF masks. The nuisance signal was regressed out to remove variation due to physiological noise and head motion using a third-order polynomial for the time series data. The de-noised time series data were band-pass filtered (0.009 < *f* <0.08 Hz) to exclude frequencies not implicated in resting state functional connectivity and then spatially smoothed using a 6-mm FWHM Gaussian filter.

### Calculating volumes of subcortical structures

We selected 10 subcortical regions (thalamus L/R, putamen L/R, hippocampus L/R, caudate L/R, and amygdala L/R) known to show regional volume atrophy in AD patients compared to normal subjects [9]. A linear registration between T1 image and MNI space was performed to map the patient’s T1 image onto the standard space for fair comparison of regional volume. Segmentations of subcortical regions were automatically performed by the FMRIB’s Integrated Registration and Segmentation Tool (FIRST) in FSL using the registered T1 image [43]. Each regional volume of subcortical region was calculated through voxel counting within segmented regions using in-house MATLAB code (Mathworks Inc., Natick, MA, USA).

### Functional network construction

**Fig 1:**
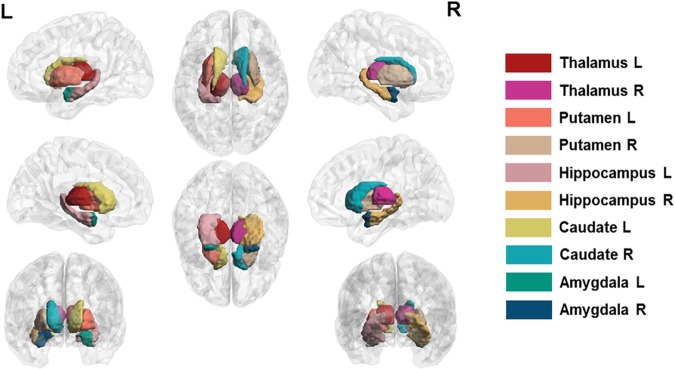
Region of interest (ROI) specifications. A total of 10 ROIs were defined as the thalamus (L/R), putamen (L/R), hippocampus (L/R), caudate (L/R), and amygdala (L/R).

We constructed individual functional networks based on correlation matrices using AFNI [41]. Network construction requires regions of interest (ROIs) to investigate correlations among brain regions These ROIs might be specified by transferring macroscopic brain structure information from a pre-defined brain atlas. We chose the Automated Anatomical Labeling (AAL) atlas to specify ROIs of the whole brain (Fig 1) [44]. Given a set of ROIs, we defined the network using nodes and edges [21–24]. Nodes were pre-defined ROIs, and edges were defined as correlation values between nodes. The edge values were entered as individual elements of a square matrix, which was referred to as the correlation matrix. We adopted un-directed and un-weighted edges for simple network construction. Fisher’s r-to-z transformation was then performed on all correlation matrices. The correlation matrix was binarized by applying a fixed-sparsity threshold. The thresholding was explored with a wide range of sparsity (1~50%). A threshold of 19%, the minimum value at which all nodes were connected in the matrices of all subjects, was adopted.

### Connectivity analysis

We quantified characteristics of functional networks using eigenvector centrality as a connectivity measure [45]. A node’s eigenvector centrality is proportional to the sum of the eigenvector centralities of all nodes directly connected to it. Nodes have high eigenvector centrality if they connect to other nodes that also have high eigenvector centrality. The centrality measure is better suited for connectivity analysis as it considers complex interplay among many brain regions with respect to a given region than using a single correlation value of a correlation matrix, which considers only the correlation of a target region with respect to a given region. The eigenvector centrality was computed at the minimum sparsity at which all ROIs were connected. Brain regions showing significant differences in eigenvector centrality among NC and MCI, NC and AD, and MCI and AD comparisons were identified within the 10 subcortical regions. In brief, we sought to find alterations in functional connectivity of NC, MCI, and AD groups through eigenvector centrality.

### Statistical tests

Group-wise differences between NC and MCI, NC and AD, and MCI and AD were explored in the 10 regions (thalamus L/R, putamen L/R, hippocampus L/R, caudate L/R, and amygdala L/R) using non-parametric permutation tests with eigenvector centrality and subcortical volumes [46]. Permutation test is an effective approach to correct p-values and address multiple comparison issue using random assignment of comparison groups [47]. Permutation tests were performed by randomly assigning subjects to three groups 5,000 times. Differences in regional volume and eigenvector centrality value were considered significant if they did not belong to the 95% of the null distribution derived from the permutation tests (p < 0.05, corrected). All statistical analyses were performed using MATLAB.

### Classification

A random forest (RF) classifier was used to distinguish among NC, MCI, and AD groups using identified regional volume and eigenvector centrality values from the permutation tests [48]. The RF classifier is an ensemble of decision-tree classifiers [49]. Each decision tree is generated by bootstrapping from the training data. To overcome the limited number of available subjects, we applied the leave-one-out-cross validation (LOOCV) for separating training and test data. For example, given 35 NC, 40 MCI, and 30 AD cases, we assigned one case as the test set and used the remaining 104 cases as the training set for the random forest classifier. The process was repeated 105 times, choosing a different test set each time. Classifier accuracy was calculated by comparing the classifier outcome with known ground truth using MATLAB.

## Results

### Regional volume differences

**Fig 2:**
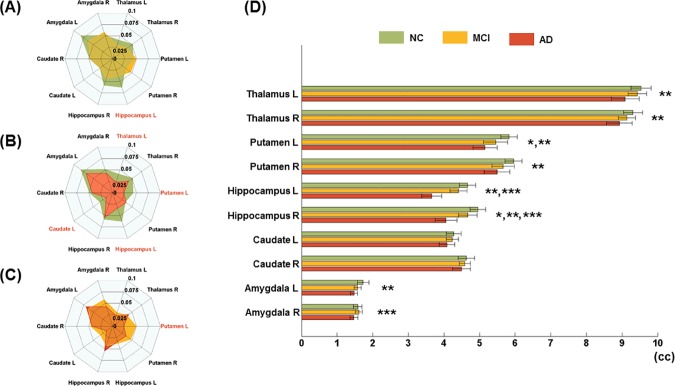
Connectional fingerprints to distinguish between comparison groups. Connectional fingerprints between (A) NC and MCI, (B) NC and AD, and (C) MCI and AD using eigenvector centrality. Regions with significant group-wise differences are marked with red text (p < 0.05). (D) Regional volumes are presented using a horizontal box plot. Regions with significant group-wise differences are marked with asterisks (*: NC and MCI, **: NC and AD, and ***: MCI and AD).

The regional volumes of 10 subcortical regions were investigated using non-parametric permutation tests with corrected p-values in order to identify significant atrophy differences between NC and MCI, MCI and AD, and NC and AD patients. Brain regions with significant (corrected p < 0.05) group-wise differences in volumes were identified. The significant regions were as follows: two regions comparing NC and MCI patients (putamen L and hippocampus R), three regions comparing MCI and AD (hippocampus L/R and amygdala R), and seven regions comparing NC and AD patients (thalamus L/R, putamen L/R, hippocampus L/R, and amygdala L). The number of regions with volumetric differences increased from 3 (between NC and MCI) to 7 (between NC and AD) as the disease progressed from NC to AD. Most regions (i.e., 9 of 10) showed a trend of decreasing volume as the disease progressed from NC to AD (Fig 2). However, the volume of amygdala R in MCI patients was larger than that of NC. Further details regarding volume differences are reported in Table 2.

**Table 2:** Regional volume values [cc] of subcortical regions (columns two, three, and four). Mean and standard deviation values are reported. Regional volume differences between comparison groups (NC/MCI, NC/AD, and MCI/AD) are reported with corrected p-values (columns five, six, and seven) for subcortical regions.

	Regional volume			Group-wise differences (Corrected p-value)		
	NC	MCI	AD	NC & MCI	NC & AD	MCI & AD
**Thalamus L**	9.54 (0.81)	9.43 (0.81)	9.09 (1.04)	0.241	**0.028**	0.064
**Thalamus R**	9.31 (0.77)	9.14 (0.76)	8.93 (0.96)	0.131	**0.035**	0.143
**Putamen L**	5.83 (0.66)	5.45 (1.06)	5.16 (0.92)	**0.020**	**0.001**	0.130
**Putamen R**	5.95 (0.70)	5.66 (0.98)	5.49 (0.97)	0.054	**0.015**	0.254
**Hippocampus L**	4.66 (0.68)	4.41 (0.77)	3.66 (0.76)	0.071	**<0.001**	**<0.001**
**Hippocampus R**	4.96 (0.64)	4.67 (0.80)	4.06 (0.83)	**0.039**	**<0.001**	**0.001**
**Caudate L**	4.27 (0.60)	4.24 (0.54)	4.08 (0.60)	0.368	0.165	0.200
**Caudate R**	4.63 (0.67)	4.59 (0.50)	4.49 (0.67)	0.386	0.247	0.306
**Amygdala L**	1.73 (0.46)	1.58 (0.32)	1.47 (0.28)	0.055	**0.002**	0.066
**Amygdala R**	1.58 (0.36)	1.61 (0.30)	1.47 (0.29)	0.669	0.078	**0.024**

### Functional connectivity differences

The eigenvector centrality of the subcortical regions was investigated using non-parametric permutation tests with corrected p-values to find significant functional connectivity differences between NC and MCI, NC and AD, and MCI and AD patients. Eigenvector centrality values were calculated at a sparsity of 19%, the minimum value at which all nodes were connected [50]. Brain regions with significant (corrected p < 0.05) group-wise differences in eigenvalue centrality were identified. The identified regions were as follows: one region comparing NC and MCI patients (hippocampus L), one region comparing MCI and AD (putamen L), and four regions comparing NC and AD patients (thalamus L, putamen L, hippocampus L, and caudate L). The left hippocampus showed lower centrality in MCI and AD group compared to the NC group. This could be interpreted as functional degeneration occurring in MCI and AD compared to NC. The left putamen also showed functional degeneration in NC and MCI compared with AD. However, the other regions did not show significant differences between any of the comparisons (NC and MCI, NC and AD, and MCI and AD). Further details regarding functional connectivity differences are reported in Table 3.

**Table 3:** Eigenvector centrality values of subcortical regions (columns two, three, and four). Mean and standard deviation values are reported. Eigenvector centrality differences between comparison groups (NC/MCI, NC/AD, and MCI/AD) are reported with corrected p-values (columns five, six, and seven) for subcortical regions.

	Eigenvector centrality			Group-wise differences (Corrected p-value)		
	NC	MCI	AD	NC & MCI	NC & AD	MCI & AD
**Thalamus L**	0.041 (0.046)	0.030 (0.040)	0.023 (0.042)	0.139	**0.032**	0.226
**Thalamus R**	0.051 (0.059)	0.037 (0.049)	0.041 (0.059)	0.125	0.233	0.613
**Putamen L**	0.043 (0.047)	0.049 (0.061)	0.023 (0.037)	0.637	**0.021**	**0.013**
**Putamen R**	0.033 (0.042)	0.047 (0.057)	0.035 (0.058)	0.878	0.534	0.166
**Hippocampus L**	0.062 (0.060)	0.037 (0.037)	0.034 (0.053)	**0.015**	**0.017**	0.324
**Hippocampus R**	0.058 (0.059)	0.043 (0.055)	0.054 (0.072)	0.115	0.545	0.849
**Caudate L**	0.032 (0.035)	0.031 (0.048)	0.016 (0.032)	0.420	**0.022**	0.061
**Caudate R**	0.048 (0.054)	0.046 (0.058)	0.043 (0.060)	0.373	0.361	0.421
**Amygdala L**	0.081 (0.064)	0.062 (0.062)	0.068 (0.075)	0.100	0.234	0.671
**Amygdala R**	0.051 (0.057)	0.058 (0.065)	0.043 (0.061)	0.716	0.240	0.124

### Classifier performance

The identified regional volume and eigenvector centrality values were used as features of the RF classifier in order to distinguish among NC, MCI, and AD cases. Features were selected if p-values comparing NC/MCI, NC/AD, and MCI/AD groups were less than 0.05 for at least two comparisons (Tables 2 and 3). Classifier accuracy was computed by comparing the classifier outcome with the ground truth of NC, MCI, and AD assignments according to criteria set by the ADNI consortium. The classifier accuracy value was achieved 53.33% for distinguishing among NC, MCI, and AD cases. The classifier chose among three possible outcomes instead of two outcomes unlike existing studies of choosing between two outcome [51].

### Connectional fingerprint

**Fig 3:**
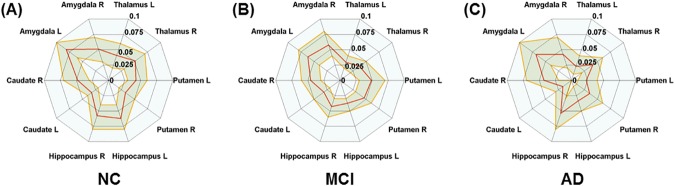
Connectional fingerprints of three groups. Connectional fingerprints of (A) NC, (B) MCI, and (C) AD using eigenvector centrality. The red line represents the mean value of the each group. The yellow line represents the 95% confidence interval.

**Fig 4:**
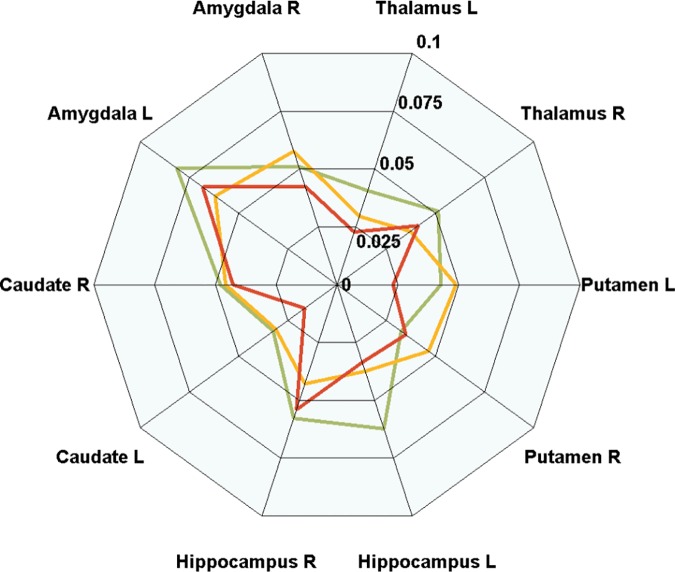
Connectional fingerprints in radar plot form. Connectional fingerprints of NC (green), MCI (yellow), and AD (red) based on eigenvector centrality. Plot of mean centrality values is given for the three groups.

The distinctive connectional information based on morphology and functional connectivity for NC, MCI, and AD groups was established in the previous section. The connectional fingerprint information is illustrated in a radar plot form in Figs 2, 3 and 4. Fig 2 shows connectional fingerprints based on regional atrophy in order to distinguish comparison groups using eigenvector centrality. Fig 3 shows connectional fingerprints of the three groups in terms of eigenvector centrality. Functional degeneration occurring in AD and MCI compared to NC was confirmed through eigenvector centrality radar plots (Fig 3). Functional degeneration increased as the disease progressed from NC to AD, as shown by decreasing centrality values in thalamus L, hippocampus L, caudate L, and caudate R (Fig 4). Furthermore, the area enclosed by the colored lines (green: NC, yellow: MCI, and red: AD) decreased as the disease progressed from NC to AD. This is another way of visualizing the impact of AD progression.

## Discussion

We confirmed volume atrophy and functional degeneration in subcortical regions using structural and functional MRI for AD and MCI patients. The functional degeneration was investigated using eigenvector centrality. Group-wise differences of regional volume and eigenvector centrality were quantified between NC and MCI, NC and AD, and MCI and AD using permutation tests. We also performed classification using the regional volume and eigenvector centrality values, which showed significant differences between comparisons. Finally, we showed connectional fingerprints of NC, AD, MCI patients using functional connectivity and regional volume atrophy of 10 subcortical regions.

Regional volume atrophy occurred in several subcortical regions. Two regions (putamen L and hippocampus R) showed significant differences in volume between NC and MCI. Seven regions (hippocampus L/R, thalamus L/R, putamen L/R, and amygdala L) showed significant differences in volume between NC and AD. Three regions (hippocampus L/R and amygdala R) showed significant differences between MCI and AD. These regions are known to be related to MCI and AD [52,53]. Thus, our results confirmed existing research. Some regions that showed significant volume differences between groups did not lead to functional degeneration in terms of eigenvector centrality. A morphological change does not necessarily imply functional connectivity change for regions, including thalamus R, putamen R, hippocampus R, amygdala L, and amygdala R. Other studies have reported similar results of no change or increase in centrality of AD/MCI compared with NC in those regions [54–56].

The eigenvector centrality showed significant differences in several regions. One region (hippocampus L) showed significant differences between NC and MCI. Four regions (thalamus L, putamen L, hippocampus L, and caudate L) showed significant differences between NC and AD. One region (putamen L) showed significant differences between MCI and AD. The left hippocampus showed a significant difference in NC vs. MCI and NC vs. AD. The left putamen showed a significant difference in NC vs. AD and MCI vs. AD. Compared to NC, AD patients showed decreased functional connectivity between the thalamus and other regions [57]. AD patients also showed greater caudate and thalamic connectivity compared with NC [58]. NC demonstrated increased connectivity of the hippocampus compared with AD [59]. Regions with decreased functional connectivity were found between the amygdala and default mode network for AD patients [52]. Thus, our functional connectivity results are largely consistent with existing research.

Connectional fingerprint information was visualized in a radar plot from, which allows intuitive integration of local and global change information. Local information was easily assessed by radial sampling of the radar plot. Global information could be easily assessed by the area enclosed by each graph line (i.e., one group). For example, the area enclosed by the lines of NC was larger than that of AD, which showed functional degeneration occurring over 10 regions as the disease progressed (Fig 4).

The RF classifier attained 53.33% accuracy in distinguishing among NC, MCI, and AD using volume and eigenvector centrality of identified regions. As the classifier had to choose from three outcomes the performance of baseline classifier is 33% unlike the binary classifier with baseline performance of 50%. Our results of three label classifier were comparable with existing studies of three label classification [60,61]. The best algorithm, developed by Sørensen et al., achieved an accuracy of 63% in the 2014 Dementia challenge [61]. Their approach considered both cortical and subcortical regions and thus had more information to work with. Our results fared worse because we focused on subcortical regions as our aim was to compute connectional fingerprint information of subcortical regions. The connectional fingerprint is succinct information to characterize the MCI and AD patients, which could serve as baseline information for future artificial intelligence (AI) related approaches. Many AI methods could benefit if there is an established feature space where comparison groups are distinctly separated.

We performed additional analyses adding posterior cingulate cortex (PCC) and precuneus regions. The results were reported in the Table C in S2 File and Table D in S2 File.

We performed the same set of functional connectivity analyses using a different atlas. Brainnetome atlas is a structural atlas with 246 sub-regions similar to AAL atlas [62]. The Brainnetome atlas has more regions than the AAL atlas and thus we had to merge a few regions into one region for some subcortical regions. For example, eight thalamus sub-regions were merged into one thalamus region in each hemisphere. Using the Brainnetome atlas, the regions with significant eigenvector centrality differences were as follows. Putamen L was found comparing NC and MCI, putamen R and hippocampus L were found comparing NC and AD, amygdala R was found comparing NC and AD, and amygdala R was found comparing MCI and AD. Eigenvector centrality values of subcortical regions using the Brainnetome atlas are reported in the Table A in S2 File. Previously using the AAL atlas, we found hippocampus L for comparing NC and MCI, putamen L for comparing MCI and AD, thalamus, putamen L, hippocampus L, and caudate L for comparing NC and AD. Results were partially consistent between using AAL and Brainnetome atlases. Two regions, hippocampus L and putamen L, were consistently found in using two atlases out of four regions (i.e., hippocampus, putamen, thalamus, and caudate). One possible reason for this result is that we adopted eigenvector centrality for assessing nodal connectivity. The eigenvector centrality of a given node is affected by the functional connectivity of all the nodes (i.e., regions) connected to the given node. Atlases might have differences how structural regions are defined. On average, a centroid of a specific region would be similar among atlases, but the periphery of a specific region might differ. These differences in specifying regions would lead to differences in nodal specification and might eventually affect eigenvector centrality. Wang et al. reported that centrality measures were unstable if different atlases were adopted [63]. They reported that 70–80% of the elements in the connectivity matrix led to low intra-class correlation coefficient (ICC) and only 20–30% of the elements in the connectivity matrix led to fair or high ICCs for comparing test and re-test reliability of different atlases.

Our study has a few limitations. First, we chose eigenvector centrality as a connectivity measure to differentiate regions in the brain network. There are other connectivity measures including degree centrality, similarity, and clustering could be jointly investigated to provide a better quantification of brain networks. Second, our study considered structural MRI and rs-fMRI. If we add another modality such as diffusion-weighted imaging, PET, or SPECT, it might provide complementary information to better quantify the functional brain network. Finally, a longitudinal study is necessary to assess the stability of our findings.

Our study reported significant brain regions that showed regional volume atrophy and functional degeneration using regional volume and eigenvector centrality for MCI and AD. A connectional fingerprint in terms of radar plot was given for NC, MCI, and AD groups to effectively visualize fingerprint information.

## Supporting information

S1 File Mean functional correlation matrix for NC, MCI, and AD groups. (ZIP)

S2 File Additional supporting information to supplement the discussion section. (DOCX)

S3 File Complete list of group authors. (PDF)
