# Supplementary material for: Structural and functional connectional fingerprints in mild cognitive impairment and Alzheimer’s disease patients
Source: PLoS One. 2017 Mar 23;12(3):e0173426. doi: 10.1371/journal.pone.0173426 (PMC5363868; doi:10.1371/journal.pone.0173426)
Supplement: S2 File — (DOCX) [file pone.0173426.s002.docx]

**Supplementary Information**

**Table A.** **Eigenvector centrality values of subcortical regions (columns two, three, and four) using the Brainnetome atlas.** Mean and standard deviation values are reported. Eigenvector centrality differences between comparison groups (NC/MCI, NC/AD, and MCI/AD) are reported with corrected p-values (columns five, six, and seven) for subcortical regions.

|  | **Eigenvector centrality** | | | **Group-wise differences  (corrected p-value)** | | |
| --- | --- | --- | --- | --- | --- | --- |
|  | **NC** | **MCI** | **AD** | **NC & MCI** | **NC & AD** | **MCI & AD** |
| **Thalamus L** | 0.027 (0.034) | 0.026 (0.034) | 0.028 (0.042) | 0.871 | 0.893 | 0.767 |
| **Thalamus R** | 0.021 (0.033) | 0.037 (0.046) | 0.037 (0.052) | 0.103 | 0.120 | 0.953 |
| **Putamen L** | 0.040 (0.048) | 0.018 (0.031) | 0.035 (0.049) | **0.018** | 0.699 | 0.078 |
| **Putamen R** | 0.049 (0.051) | 0.045 (0.055) | 0.025 (0.041) | 0.739 | **0.045** | 0.106 |
| **Hippocampus L** | 0.015 (0.031) | 0.023 (0.031) | 0.036 (0.048) | 0.275 | **0.040** | 0.167 |
| **Hippocampus R** | 0.026 (0.040) | 0.028 (0.038) | 0.035 (0.052) | 0.858 | 0.412 | 0.458 |
| **Caudate L** | 0.032 (0.041) | 0.038 (0.048) | 0.033 (0.039) | 0.563 | 0.952 | 0.618 |
| **Caudate R** | 0.035 (0.047) | 0.027 (0.041) | 0.044 (0.055) | 0.421 | 0.448 | 0.133 |
| **Amygdala L** | 0.011 (0.017) | 0.023 (0.038) | 0.021 (0.041) | 0.092 | 0.198 | 0.865 |
| **Amygdala R** | 0.043 (0.053) | 0.046 (0.051) | 0.016 (0.035) | 0.804 | **0.020** | **0.007** |

**Table B. Concentrations of Aβ_1-42_ in NC, MCI, and AD.** Mean and standard deviation values are reported. Aβ_1-42_ differences between comparison groups (NC/MCI, NC/AD, and MCI/AD) are reported with p-values (columns four, five, and six).

| **Aβ_1-42_ (*pg*/*mL*)** | | | **Group-wise differences (p-value)** | | |
| --- | --- | --- | --- | --- | --- |
| **NC** | **MCI** | **AD** | **NC & MCI** | **NC & AD** | **MCI & AD** |
| 187.63 (52.16) | 178.13 (52.25) | 130.27 (20.80) | 0.492 | **< 0.001** | **< 0.001** |

**Discussion related to Tables C and D**

We performed additional analyses adding posterior cingulate cortex (PCC) and precuneus regions. We applied the same procedures to measure regional volume of two additional structures using FreeSurfer as the FSL did not provide volumetry results for the two mentioned structures^1^. PCC L, PCC R, precuneus L, and precunus R showed significant regional volume differences for comparisons of NC/AD and MCI/AD. The four regions did not show significant volume differences for NC/MCI comparison. For the PCC and precuneus regions, it might imply that volume atrophy occurs during the transition from MCI to AD. We also compared eigenvector centrality differences of PCC and precuneus regions for NC, MCI, and AD groups. There were no significant functional connectivity differences as shown in S4 Table. We also quantified the performance of the RF classifier using additional volume measures of PCC and precuneus regions. The RF classifier achieved accuracy of 48.57%, which is lower than the accuracy of 53.33% when only subcortical features were considered. The additional volume measurements of cortical region might provide contradictory information to the RF classifier, which might lead to lower performance. Further investigation is needed what causes the RF classifier to perform worse when additional information is given.

[1] Fischl, B. FreeSurfer. *Neuroimage* **62,** 774–781 (2012).

**Table C. Regional volume values [cc] of PCC and precuneus regions (columns two, three, and four).** Mean and standard deviation values are reported. Regional volume differences between comparison groups (NC/MCI, NC/AD, and MCI/AD) are reported with corrected p-values (columns five, six, and seven) for subcortical regions.

|  | **Regional volume** | | | **Group-wise differences (corrected p-value)** | | |
| --- | --- | --- | --- | --- | --- | --- |
|  | **NC** | **MCI** | **AD** | **NC & MCI** | **NC & AD** | **MCI & AD** |
| **PCC L** | 3.77 (0.55) | 3.80 (0.53) | 3.41 (0.47) | 0.738 | **0.006** | **0.001** |
| **PCC R** | 3.87 (0.59) | 3.98 (0.70) | 3.48 (0.54) | 0.491 | **0.007** | **0.002** |
| **Precuneus L** | 10.56 (1.26) | 10.36 (1.36) | 8.97 (1.25) | 0.527 | **< 0.001** | **< 0.001** |
| **Precuneus R** | 10.75 (1.24) | 10.44 (1.23) | 9.77 (1.39) | 0.282 | **0.004** | **0.036** |

**Table D. Eigenvector centrality values of PCC and precuneus regions (columns two, three, and four).** Mean and standard deviation values are reported. Eigenvector centrality differences between comparison groups (NC/MCI, NC/AD, and MCI/AD) are reported with corrected p-values (columns five, six, and seven) for subcortical regions.

|  | **Eigenvector centrality** | | | **Group-wise differences (corrected p-value)** | | |
| --- | --- | --- | --- | --- | --- | --- |
|  | **NC** | **MCI** | **AD** | **NC & MCI** | **NC & AD** | **MCI & AD** |
| **PCC L** | 0.036 (0.043) | 0.039 (0.051) | 0.029 (0.041) | 0.636 | 0.238 | 0.174 |
| **PCC R** | 0.035 (0.042) | 0.043 (0.051 | 0.051 (0.061) | 0.782 | 0.870 | 0.685 |
| **Precuneus L** | 0.037 (0.048) | 0.042 (0.052) | 0.053 (0.072) | 0.594 | 0.887 | 0.832 |
| **Precuneus R** | 0.051 (0.056) | 0.037 (0.049) | 0.052 (0.070) | 0.145 | 0.589 | 0.898 |
